# Frequency Evaluation of the *Interleukin-6* −174G>C Polymorphism and *Homeostatic Iron Regulator (HFE)* Mutations as Disease Modifiers in Patients Affected by Systemic Lupus Erythematosus and Rheumatoid Arthritis

**DOI:** 10.3390/ijms242216300

**Published:** 2023-11-14

**Authors:** Mattia Carini, Micaela Fredi, Ilaria Cavazzana, Roberto Bresciani, Fabiana Ferrari, Eugenio Monti, Franco Franceschini, Giorgio Biasiotto

**Affiliations:** 1 Department of Molecular and Translational Medicine, University of Brescia, 25123 Brescia, Italy; m.carini001@unibs.it (M.C.); roberto.bresciani@unibs.it (R.B.); eugenio.monti@unibs.it (E.M.); 2 Highly Specialized Laboratory, ASST Spedali Civili di Brescia, Piazzale Spedali Civili 1, 25123 Brescia, Italy; 3 Department of Clinical and Experimental Sciences, University of Brescia, 25123 Brescia, Italy; micaela.fredi@unibs.it (M.F.); franco.franceschini@unibs.it (F.F.); 4 Rheumatology and Clinical Immunology Unit, ASST Spedali Civili di Brescia, Piazzale Spedali Civili 1, 25123 Brescia, Italy; ilariacava@virgilio.it; 5 Pediatrics, Mother’s and Baby’s Health Department, Poliambulanza Foundation Hospital Insitute, 25124 Brescia, Italy; fabiana.ferrari@poliambulanza.it

**Keywords:** *HFE*, *IL-6*, iron metabolism, inflammation, systemic lupus erythematosus, rheumatoid arthritis

## Abstract

Autoimmune diseases are generally characterized by a multifactorial etiology and are often associated with a genetic predisposition. Both iron metabolism and the inflammatory cytokine system have been shown to play a pivotal role in the dysregulation of the immune response in many different autoimmune conditions, rheumatologic diseases included. The purpose of this work was to analyze the frequency of mutations altering the expression of IL-6 or influencing iron metabolism in patients affected by autoimmune diseases such as Rheumatoid Arthritis (RA) and Systemic Lupus Erythematosus (SLE). In this study, 144 patients were enrolled: 77 and 67 patients were affected by RA and SLE, respectively. In these cohorts, the frequency of the *IL-6* polymorphism −174G>C located in the *IL-6* gene promoter was tested. Moreover, the frequencies of the three *HFE* gene variations associated with iron overload were analyzed: p.His63Asp, p.Ser65Cys and p.Cys282Tyr. The two mutations p.His63Asp and p.Ser65Cys in the *HFE* gene did not reach statistical significance in any of the comparisons, regardless of the statistical model, cohorts of patients and control populations analyzed. The frequencies of the p.Cys282Tyr mutation and the *IL-6* polymorphism −174G>C were found to be overall significantly decreased in RA and SLE patients when the Dominant model and Allele contrast were adopted with both the Odds Ratio and Chi-square. Although further investigation is needed, the examination of the frequencies of the −174G>C *IL-6* promoter polymorphism and *HFE* mutations may add some valuable information on the interplay linking iron metabolism, inflammation and immunity in autoimmune diseases such as SLE and RA.

## 1. Introduction

The immune system consists of two main lines of defense: innate and adaptive immunity. While the innate response is non-specific, the adaptive response is antigen-specific and is responsible for maintaining a delicate balance between containing infections and avoiding self-reactions that could trigger autoimmune diseases [1]. In addition to ensuring defense against various pathogens and helping to eliminate malignant cells, the immune system cooperates in maintaining metabolic homeostasis in the human organism. This is mainly because micro-organisms need metabolic products to replicate in the human body, and, among these, iron is particularly important. Consequently, one of the strategies employed by the innate immune system to contain infections is to deprive pathogens of available iron through intracellular sequestration. This mechanism mainly depends on an increase in hepcidin production under the stimulus of inflammatory cytokines such as Interleukin-6 (IL-6) in tissues and in the blood, and its most evident consequence is the development of anemia of inflammation [2]. On the other hand, evidence has shown that the proteins involved in iron metabolism may also influence the complex adaptive immunity. A key role is played by the Homeostatic Iron Regulator protein (HFE), which is structurally similar to Major Histocompatibility Complex I (MHC I) molecules and has been demonstrated to shape the adaptive response by unbalancing or affecting the expression of T-cell subpopulations [3]. Consequently, this protein might also contribute to autoimmune disease and to immunological conditions in which the adaptive response is unbalanced per se. In addition to this, HFE is responsible for the iron-sensing mechanism, which regulates iron absorption and its utilization in the human body [4]. Mutations in the *HFE* gene are known to be usually involved in hereditary forms of iron overload, which are generally named hemochromatosis. The main mutations associated with this disorder are rs1799945 (p.His63Asp), rs1800730 (p.Ser65Cys) and rs1800562 (p.Cys282Tyr). To date, this genetic condition is classified as “Hemochromatosis related to *HFE*”, which has replaced the previous outdated definition, “type I hereditary hemochromatosis” [5].

Two of the principal autoimmune diseases in which the unbalancing of the adaptive immune response plays a crucial part are Rheumatoid Arthritis (RA) and Systemic Lupus Erythematosus (SLE). RA is an autoimmune disease whose main clinical sign is symmetric chronic inflammatory synovitis, which may produce bone erosions and disability. This condition is also characterized by systemic involvement, which can generate multiple-organ failure and result in death in the worst cases [6]. Although the event that triggers autoimmune inflammation is still uncertain, a key role in adaptive immune dysregulation is played by inflammatory cytokines, both in the onset and in the evolution of the disease [7,8]. Evidence has shown that IL-6 and its soluble receptor are crucial in this process, and their serum levels were found to be higher in patients affected by RA when compared to those in the healthy population [9]. Moreover, IL-6 is the target of two biological drugs approved for the treatment of RA (tocilizumab and sarilumab), emphasizing the key role of this interleukin in the activity of the disease. Similarly, SLE is a chronic autoimmune disease with a heterogeneous systemic clinical presentation and characterized by immune complex deposition and immune dysregulation [10]. This condition is characterized by the unbalancing of both the innate and adaptive lines of the immune system and may cause a strong inflammatory response that can damage almost every organ or tissue, with various clinical manifestations, depending on the organ system affected [11]. In addition, one of its features is the abnormal production of antinuclear or cytoplasmatic autoantibodies. Increased serum levels of IL-6 were detected in SLE patients as well, and even though its role in disease activity is still uncertain, this has been sufficient to confirm its role in SLE pathogenesis [12,13].

In fact, the expression of IL-6 has been correlated with numerous other pathological conditions, such as infections, neoplasms and autoimmune diseases [14]. It can be produced by various tissues and cells, lymphocytes included, and it is implicated in inflammation, immunity and tissue regeneration [15]. This cytokine is encoded by a relatively small gene of 5 kb composed of five exons and located on the short arm of chromosome 7 [16]. Recently, the polymorphism rs1800795 (c. −174G>C) in the promoter region of the *IL-6* gene has been reported to influence the level of expression of this cytokine, and it has been investigated as a risk factor in association with many diseases [17]. Moreover, this polymorphism has been reported as a modifier variant in patients affected by hemochromatosis related to *HFE*, in which the homozygous genotype CC is significantly correlated with a higher degree of iron overload [18]. This observation further highlights the possible connection between iron metabolism and immunity.

Given the key role played by IL-6 in autoimmunity, the main purpose of this work was to analyze the frequency of the polymorphism rs1800795 (c. −174G>C) in the *IL-6* gene promoter in two cohorts of patients affected by RA and SLE. In addition, we also tried to investigate the frequencies of three mutations in the *HFE* gene involved in impaired iron metabolism.

## 2. Results

**Figure 1:**
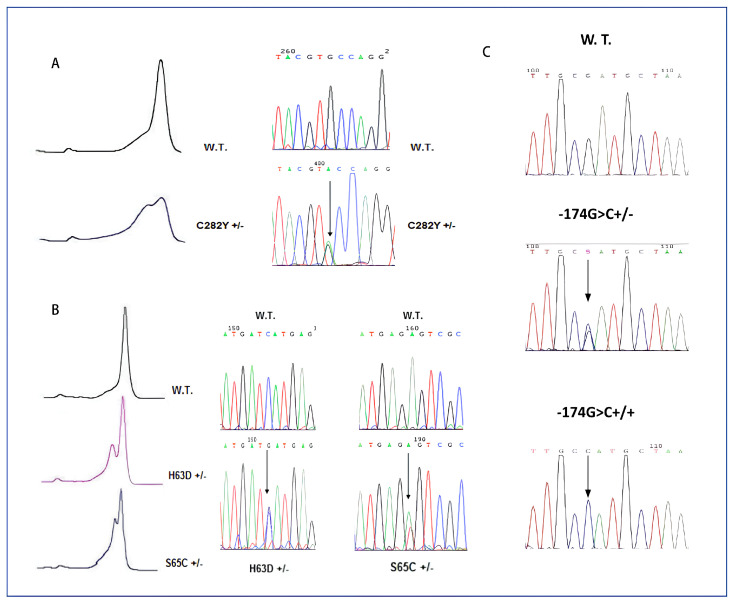
DHPLC profiles and electropherograms via Sanger sequencing of the mutations analyzed are reported: (**A**) p.Cys282Tyr mutation in *HFE* exon 4; (**B**) p.His63D and p.Cys65Ser mutations in *HFE* exon 2; (**C**) c. −174G>C polymorphism in promoter of *IL-6* gene.

In this study, 77 RA and 69 SLE patients were enrolled, and in these cohorts, the polymorphism rs1800795 (−174G>C), located in the promoter region of *IL-6*, was studied. In addition, we analyzed the frequencies of the DNA variations rs1799945 (c.187C>G; p.His63Asp) and rs1800730 (c.193A>T; p.Ser65Cys) in exon 2 and rs1800562 (c.845G>A; p.Cys282Tyr) in exon 4 of the *HFE* gene (Figure 1). The frequencies obtained in the studied cohorts were compared with those reported in public databases, such as gnomAD and 1000G, using only European (non-Finnish) data, with the aim of having two populations of controls of the same ethnicity as the patients. To enhance the genetic background similarity between patients and controls, we used data from the Tuscan population in the European (non-Finnish) cohort of 1000 G, despite the small sample. Finally, the cumulative cohort for RA + SLE patients was compared to the same populations of controls.

As expected, interesting results were obtained when investigating the frequency of the polymorphism rs1800795 (−174G>C), which was reported to influence the transcription of the gene coding for the inflammatory cytokine IL-6. The testing of this polymorphism showed 4 homozygous and 26 heterozygous carriers in the SLE population (respectively, 6% and 38,8% of the total), while the analysis in the RA cohort revealed 9 homozygous and 34 heterozygous cases (respectively, 11.6% and 44.2% of the total). When analyzing the SLE cohort in comparison to the gnomAD population, a decrease in the frequency of genotypes containing the C allele was observed, and the tests were statistically significant using both the Dominant model (CC + CG vs. GG; Odds Ratio (OR) = 0.409, 0.2525–0.662; *p* = 0.0003; Chi-square *p* = 0.0003) and Allele contrast (C vs. G; OR = 0.5017; 0.3423–0.7353; *p* = 0.0004; Chi-square *p* = 0.0003). The same result was obtained comparing SLE patients with the 1000G population using both the Dominant model (OR = 0.4558, 0.2724–0.7626; *p* = 0.0028) and the Allele contrast (OR = 0.5167, 0.3457–0.7725; *p* = 0.0013; Chi-square *p* = 0.0011). Conversely, the comparison with the Tuscan population only showed a trend toward significance (Allele dominant, OR, *p* = 0.0915; Chi-square, *p* = 0.874; Allele contrast, OR, *p* = 0.0938; Chi-square, *p* = 0.0929). As for the RA cohort, statistical significance was reached when using the Allele contrast model versus the gnomAD population (OR = 0.6963, 0.4984–0.9727; *p* = 0.0338; Chi-square *p* = 0.0329), confirming the reduction in the frequency of allele C, while the comparison with the 1000G database using the same model showed only a trend toward significance (OR, *p* = 0.0677; Chi-square, *p* = 0.0669). Despite the different role played by IL-6 in SLE when compared to RA, in consideration of the low number of subjects enrolled, we decided to combine the RA and SLE populations into a single cohort representative of the overall sample of patients affected by autoimmune disease in general. As a result, the comparison of the RA+SLE cohort with the gnomAD database reached statistical significance when using both the statistical models employed (Dominant model; OR = 0.5186, 0.0.3738–0.7195; *p* = 0.0001; Chi-square *p* ≤ 0.0001; Allele contrast; OR = 0.5815; 0.4516–0.7478; *p* ≤ 0.0001; Chi-square *p* ≤ 0.0001). The statistical significance was also confirmed when comparing it with the 1000G population (Dominant model; OR = 0.5779, 0.3976–0.8401; *p* = 0.0041; Chi-square *p* ≤ 0.0023; Allele contrast; OR = 0.5989; 0.4518–0.7938; *p* = 0.0004; Chi-square *p* = 0.0003). All of these results are reported in Table 1.

The analysis of the *HFE* mutation rs1800562 (c.845G>A p.Cys282Tyr) showed one heterozygous patient in the SLE cohort and two heterozygous patients in the RA cohort (respectively, 1.5% in SLE and 2.6% in RA). When the SLE cohort was compared to the European (non-Finnish) population in the gnomAD database, the analysis showed a statistically significant decrease in this variation when the Dominant model (AA + GA vs. GG) was adopted with both the OR (OR = 0.1207, 95% CI 0.0167–0.8696; *p* = 0.0359) and Chi-square (*p* = 0.0426). The same results were obtained by using the Allele contrast model (A vs. G), where the OR remained statistically significant (OR = 0.1219, 95% CI 0.0172–0.8790; *p* = 0.0368) and Chi-square further decreased (*p* = 0.0127). When the SLE cohort was compared to the Tuscan 1000G population using the Allele contrast, only the Chi-square value reached statistical significance (*p* = 0.0417). The comparison with the other populations showed a trend toward significance but failed to reach the threshold value. In particular, the comparison with the 1000G population using the Allele contrast model showed a Chi-square value very close to the significance cut-off (*p* = 0.0593). When the RA cohort was compared to the gnomAD population, the analysis showed statistical significance only when using the Dominant model with the OR test (OR = 0.2124, 95% CI 0.0521–0.8652; *p* = 0.0306), while the Chi-square result was just above the threshold value (*p* = 0.0578). When the Allele contrast model was used, both tests reached significance (OR = 0.1229, 95% CI 0.0172–0.8790; *p* = 0.0368; Chi-Square *p* = 0.0174). The comparative analysis using the other populations remained near the cut-off (*p* values ranged between *p* = 0.1098 and *p* = 0.0722), except for the Tuscan cohort, where the Chi-square test using the Dominant model produced results far from the threshold (*p* = 0.1867). Curiously, the results obtained by comparing SLE+RA patients to the three control populations were statistically significant, regardless of the model employed (Table 2).

The study of the frequency of the variation rs1799945 (c.187C>G p.His63Asp) showed 1 homozygous (1.5% of the total) and 15 heterozygous (22.4% of the total) patients in the SLE cohort and 26 heterozygous carriers (33.8% of the total) in the RA population. The study of rs1800730 (c.193A>T p.Ser65Cys) showed only one heterozygous case (1.5% of the total) in SLE patients. These two mutations in the *HFE* gene did not reach statistical significance in any of the comparisons, regardless of the statistical model, cohorts of patients and control populations used (Table 3 and Table 4).

## 3. Discussion

Autoimmune diseases are generally characterized by an unspecified etiology and may be caused by both genetic and environmental factors. Whereas in SLE, it is difficult to separate the contribution of the genetic background from environmental factors [19], more emphasis has been attributed to environmental triggers for RA [20]. To clarify this complex scenario, the study of genetic predisposition can be very useful for understanding the various clinical phenotypes of the patients.

The rs1800795 (−174G>C) polymorphism consists of a base change from guanine (G) to cytosine (C) in the promoter region of the *IL-6* gene. When the C allele is present in the promoter, lower levels of the IL-6 transcript are produced, and IL-6 is decreased in tissues and peripheral blood. On the contrary, the G allele is related to the increased production of this cytokine and a higher risk of inflammatory disorders [17,21,22].

The study of this polymorphism showed a statistically significant decrease in the C allele when using either the Chi-square or OR when the SLE cohort was compared with gnomAD and 1000G populations by applying both the Dominant model and Allele contrast. The comparison with the Tuscan 1000 G population only showed a decreasing trend without reaching statistical significance. The role of this polymorphism in SLE predisposition has been debated in the literature, and different distributions of the allele frequency depending on ethnicity were proposed by some authors [23,24,25,26]. In our SLE cohort, a statistically significant increase in the G allele was found, which is coherent with the higher production of IL-6 found in some Caucasian populations [24,26,27].

When RA patients were considered, significant values were obtained only when using the Allele contrast model versus the gnomAD population with both the Chi-square and *OR*. These results of a partial association are in line with the heterogeneous observations obtained when this polymorphism was investigated in different RA case studies and when meta-analyses were performed [28,29]. Recently, the rs1800795 (−174G>C) polymorphism was associated with an augmented risk of RA mainly in the Asian population, while the correlation in the Caucasian population is still unclear [17,30].

Bearing in mind the different implications of IL-6 expression in the pathogenesis of RA and SLE and, therefore, that this was only an attempt to partially represent autoimmune diseases in general, these results were confirmed by combining RA and SLE patients into a unique cohort.

When taking the *HFE* gene into account, the scenario seemed more complex. It was in 1976 that Simon and Fauchet evaluated the correlation between HLA and a non-immunological disease, such as idiopathic hemochromatosis. The results of this work were published in a *Lancet* paper, which surprisingly paved the way for new research on the relationship between iron metabolism and immunology [31]. Twenty years later, Feder et al. discovered a new MHC class I-like gene strongly associated with familial hemochromatosis: the *HFE* gene [32]. Further studies better defined *HFE* as a non-classical MHC Ib molecule, but its antigen-binding capacity was not clearly demonstrated [33]. Despite not being able to bind peptides, *HFE* was shown to affect the set-up of the T-cell repertoire and, in particular, the CD4/CD8 ratio, which was found to be unbalanced in hemochromatosis patients carrying *HFE* mutations [34,35].

To evaluate the contribution of *HFE* gene variations in autoimmune disease, the frequencies of the three most common mutations were studied in two cohorts of RA and SLE patients, which were compared with two European and one Italian control group taken from available public databases (gnomAD and 1000G). The analysis of the mutations rs1799945 (c.187C>G p.His63Asp) and rs1800730 (c.193A>T p.Ser65Cys) did not show any differences from the populations in the databases. In fact, the role of these mutations is debated in the literature: while rs1800730 (c.193A>T p.Ser65Cys) is characterized by a low minor allele frequency (MAF) and was not previously associated with RA or LES, the role of rs1799945 (c.187C>G p.His63Asp) is unclear. Some authors found an association between this variation and RA [36,37], while others did not find a correlation, despite the large number of carriers examined [38]. In the present study, this mutation was not prevalent in the analyzed cohorts, but considering the small number of patients enrolled, the number of cases should be increased, possibly with the inclusion of information on the ethnicity of the subjects. In our cohorts, none of the patients carrying *HFE* mutations received a diagnosis of hemochromatosis or was tested by liver biopsy for iron overload.

Conversely, the study of the mutation rs1800562 (c.845G>A p.Cys282Tyr) showed a statistically significant decrease in the frequency of the variation calculated when using either the OR or Chi-square model in the SLE cohort when compared with the gnomAD population using both the Dominant model and Allele contrast. The comparisons with the other populations only showed a trend toward significance, with the exception of the Tuscan 1000G population, where the Allele contrast model was statistically significant according to the Chi-square. The analysis of the RA cohort versus the gnomAD population reached statistical significance with all of the models applied, except for when the Chi-square test result was obtained by using the Dominant model (*p* = 0.0578). The SLE + RA cohort was statistically significant when compared with all of the control populations employed. Curiously, the p.Cys282Tyr mutation is the main mutation responsible for hereditary hemochromatosis (HH) and is characterized by incomplete penetrance [5,39]. Not much has been reported in the literature about this mutation and autoimmune diseases. For example, even though the p.Cys282Tyr mutation has not been clearly associated with multiple sclerosis, some authors have hypothesized that it might play a role in anticipating the onset of the disease and in the worsening of the clinical presentation [40,41,42]. A study on homozygous p.Cys282Tyr patients affected by HH reported that autoimmune conditions were common in these carriers [43]. This study found that 35 patients out of the 235 patients analyzed (about 15%) were affected by autoimmune disorders. Most of these subjects were affected by Hashimoto’s thyroiditis (19 patients; 8% of the total), and one of these patients was also affected by SLE. Even though four more patients were affected by RA (1.7% of the total), the authors noted that the percentage did not differ from that of the U.S. general population. Therefore, HH patients did not seem to be affected by SLE and RA more than the average population [43]. In addition, since hemochromatosis can cause a form of hand arthropathy similar to that of RA, specific evaluations should be performed in order to make the correct diagnosis [44].

As reported above, the frequency of the p.Cys282Tyr mutation was found to be decreased in AR RA and SLE when compared with the available databases, and the data were also confirmed by analyzing all patients affected by an autoimmune disease in a unique cohort. Considering the small number of patients enrolled in both the RA and SLE cohorts and the relatively low minor allele frequency of this mutation (MAF = 0.039, gnomAD), these data should be confirmed by expanding the cohorts in the future. Nevertheless, based on these preliminary observations, one could hypothesize a protective role for this mutation. The p.Cys282Tyr protective effect could be due to the decreased efficiency of the iron sensor complex responsible for facilitating iron absorption via hepcidin production in response to iron elevation in peripheral blood [45], thus improving iron storage and mitigating the state of iron deficiency in SLE. In fact, SLE patients suffer from reduced iron stores and insufficient iron availability due to absolute and functional iron deficiency, which can cause mitochondrial dysfunction and ultimately affect the CD4^+^ and CD8^+^ T-cell functionality, among other metabolic pathways [46,47]. In contrast, the association between rs1800562 (c.845G>A p.Cys282Tyr) and RA has been much discussed in the literature [36,37,48]. It is interesting to note that Pilling et al. found a correlation between p.Cys282Tyr homozygous carriers and RA in men. This correlation was not observed in women, where the cases of RA seemed to be slightly less represented than in controls [38]. The strong prevalence of women in this study (58 females of 77 RA patients) could explain the decrease in the prevalence of p.Cys282Tyr mutated RA subjects, but this observation must be confirmed by expanding the case studies.

## 4. Materials and Methods

### 4.1. Genetic Analyses

Genomic DNA was obtained from EDTA peripheral blood using the Wizard Genomic DNA Purification kit (Promega, Madison, WI, USA) as required by the manufacturer’s instructions. The DNA samples were quantified using a Qubit 2.0 Fluorometer (Thermo Fisher Scientific, Waltham, MA, USA) by means of the Qubit dsDNA Hs assay kit (Thermo Fisher Scientific).

The amplification and denaturation of the fragments of exons 2 and 4 of the *HFE* gene were performed, respectively, by PCR and HPLC analysis (Transgenomic Inc., Omaha, NE, USA), as reported in ref. [49,50], in order to detect the following mutations: rs1799945 (p.His63Asp), rs1800730 (p.Ser65Cys) and rs1800562 (p.Cys282Tyr). The polymorphism rs1800795 (−174G>C) located in the promoter region of the *IL-6* gene was amplified using specifically designed primers and directly analyzed by Sanger sequencing in consideration of the high frequency of this variation in the European population. Sanger sequencing was conducted with an ABI 3500 Genetic Analyzer (Thermo Fisher Scientific), using GeneScan-500LIZ (Thermo Fisher Scientific) as the internal size standard.

### 4.2. Statistical Analyses

The following genetic comparison models were used for statistical analyses: Dominant models for rs1799945 (DD + HD vs. HH), rs1800730 (CC + CS vs. SS), rs1800562 (YY + YC vs. CC) and rs1800795 (CC + CG vs. GG) and Allele contrasts for rs1799945 (D vs. H), rs1800730 (C vs. S), rs1800562 (Y vs. C) and rs1800795 (C vs. G).

The Chi-square test was applied to compare the expected and observed frequencies of the variations in the two cohorts of patients and the same variations obtained from the public databases gnomAD and 1000G as controls. Only the European (non-Finnish) population data in gnomAD and 1000G and the Tuscan (Italian) population in 1000G were considered. In addition, the Odds Ratio (OR) with a 95% confidence interval (95% CI) was calculated for all comparisons. The *p* values obtained using both Chi-square and OR were considered significant when less than 0.05 (*p* < 0.05).

## 5. Conclusions

In conclusion, examining the frequencies of polymorphisms and mutations in possible modifier genes is complex but essential for an understanding of the molecular mechanisms of multifactorial diseases, immunological conditions included. Clearly, among the factors that could interfere with their heterogeneous clinical manifestations, a role is played not only by genetic variability but also by ethnicity and environmental factors. Notwithstanding this, the investigation of the main *HFE* mutations and the −174G>C polymorphism in the *IL-6* promoter may add some information on the interplay linking iron metabolism, inflammation and immunity in autoimmune diseases such as SLE and RA.

## 6. Patients

We studied 77 patients (19 males and 58 females) affected by RA and 67 patients (2 males and 65 females) affected by SLE, consecutively enrolled in a year at the Rheumatology Unit of Spedali Civili of Brescia/University of Brescia. The patients were assessed on the basis of clinical presentation and laboratory results and monitored in the follow-up period, in accordance with the European Alliance of Associations for Rheumatology/American College of Rheumatology (EULAR/ACR) standards for RA and the Systemic Lupus Erythematosus International Collaborating Clinic Group (SLICC) criteria for SLE [51,52,53]. The patients were then evaluated and treated according to standard guideline recommendations.

## Figures and Tables

**Table 1:** The rs1800795 mutation (IL6, c. −174G>C) in the LES cohort, RA cohort and cumulative autoimmune cohort compared with the population in gnomAD and 1000G databases (the genotypes and correlations reaching statistical significance are indicated **in bold**).

Population	Genotype	Odds Ratio				Chi-Square
		OR	95% CI	Z Score	*p* Value	*p* Value
SLE vs. EU gnomAD	**CC + CG vs. GG**	**0.409**	**0.2525, 0.6623**	**3.635**	**0.0003**	**0.0003**
	**C vs. G**	**0.5017**	**0.3423, 0.7353**	**3.536**	**0.0004**	**0.0003**
SLE vs. EU 1000G	**CC + CG vs. GG**	**0.4558**	**0.2724, 0.7626**	**2.992**	**0.0028**	**0.0026**
	**C vs. G**	**0.5167**	**0.3457, 0.7725**	**3.219**	**0.0013**	**0.0011**
SLE vs. Tuscany 1000G	CC + CG vs. GG	0.5885	0.3179, 1.0894	1.688	0.0915	0.0874
	C vs. G	0.667	0.4154, 1.0711	1.676	0.0938	0.0929
RA vs. EU GnomAD	CC + CG vs. GG	0.6379	0.4066, 1.0008	1.957	0.0504	0.1000
	**C vs. G**	**0.6963**	**0.4984, 0.9727**	**2.122**	**0.0338**	**0.0329**
RA vs. EU 1000G	CC + CG vs. GG	0.7109	0.4376, 1.1549	1.378	0.1681	0.1989
	C vs. G	0.7171	0.5020, 1.0246	1.827	0.0677	0.0669
RA vs. Tuscany 1000G	CC + CG vs. GG	0.9179	0.5082, 1.6581	0.284	0.7765	0.9414
	C vs. G	0.9257	0.5987, 1.4313	0.347	0.7284	0.6984
Overall vs. EU gnomAD	**CC + CG vs. GG**	**0.5186**	**0.3738, 0.7195**	**3.930**	**0.0001**	**<0.0001**
	**C vs. G**	**0.5815**	**0.4516, 0.7478**	**4.203**	**<0.0001**	**<0.0001**
Overall vs. EU 1000G	**CC + CG vs. GG**	**0.5779**	**0.3976, 0.8401**	**2.873**	**0.0041**	**0.0023**
	**C vs. G**	**0.5989**	**0.4518, 0.7938**	**3.566**	**0.0004**	**0.0003**
Overall vs. Tuscany 1000G	CC + CG vs. GG	0.7463	0.4508, 1.2353	1.138	0.2251	0.4101
	C vs. G	0.7731	0.5303, 1.127	1.338	0.1807	0.1803

**Table 2:** The rs1800562 mutation (*HFE*, NM_000410.4, c.845G>A, p.Cys282Tyr) in the LES cohort, RA cohort and cumulative autoimmune cohort compared with the population in GnomAD and 1000G databases (the genotypes and correlations reaching statistical significance are indicated **in bold**).

Population	Genotype	Odds Ratio				Chi-Square
		OR	95% CI	Z Score	*p* Value	*p* Value
SLE vs. EU gnomAD	**AA + GA vs. GG**	**0.1207**	**0.0167, 0.8696**	**2.099**	**0.0359**	**0.0426**
	**A vs. G**	**0.1219**	**0.0172, 0.8790**	**2.089**	**0.0368**	**0.0127**
SLE vs. EU 1000G	AA + GA vs. GG	0.1754	0.0237,1.2972	1.705	0.0882	0.1575
	A vs. G	0.1816	0.0248, 1.3318	1.678	0.0933	0.0593
SLE vs. Tuscany 1000G	AA + GA vs. GG	0.1470	0.0184, 1.1756	1.807	0.0707	0.1164
	**A vs. G**	0.1534	0.0194, 1.2122	1.778	0.0755	**0.0417**
RA vs. EU gnomAD	**AA + GA vs. GG**	**0.2124**	**0.0521, 0.8652**	**2.160**	**0.0306**	0.0578
	A vs. G	**0.1229**	**0.0172, 0.8790**	**2.089**	**0.0368**	**0.0174**
RA vs. EU 1000G	AA + GA vs. GG	0.3087	0.0731, 1.304	1.599	0.1098	0.0941
	A vs. G	0.1816	0.0248, 1.3318	1.678	0.0933	0.0976
RA vs. Tuscany 1000G	AA + GA vs. GG	0.2587	0.0550, 1.2116	1.712	0.0868	0.1867
	A vs. G	0.1534	0.0194, 1.2122	1.778	0.0755	0.0722
Overall vs. EU gnomAD	**AA + GA vs. GG**	**0.1695**	**0.054, 0.5319**	**3.042**	**0.0024**	**0.0025**
	**A vs. G**	**0.1720**	**0.0551, 0.5367**	**3.032**	**0.0024**	**0.0006**
Overall vs. EU 1000G	**AA + GA vs. GG**	**0.2463**	**0.0750, 0.8082**	**2.311**	**0.0208**	**0.0447**
	**A vs. G**	**0.2542**	**0.0781, 0.8279**	**2.274**	**0.0230**	**0.0143**
Overall vs. Tuscany 1000G	**AA + GA vs. GG**	**0.2064**	**0.0554, 0.7694**	**2.35**	**0.0188**	**0.0368**
	**A vs. G**	**0.2147**	**0.0584, 0.7901**	**2.315**	**0.0206**	**0.0113**

**Table 3:** The rs1799945 mutation (*HFE*, NM_000410.4, c.187C>G, p.His63Asp) in the LES cohort, RA cohort and cumulative autoimmune cohort compared with the population in gnomAD and 1000G databases.

Population	Genotype	Odds Ratio				Chi-Square
		OR	95% CI	Z Score	*p* Value	*p* Value
SLE vs. EU gnomAD	GG + GC vs. CC	0.8641	0.4923, 1.5156	0.5100	0.6103	0.8430
	G vs. C	0.8161	0.4840, 1.3760	0.762	0.4458	0.4128
SLE vs. EU 1000G	GG + GC vs. CC	0.7044	0.3894, 1.2741	1.159	0.2465	0.4276
	G vs. C	0.6529	0.3777, 1.1285	1.527	0.1268	0.1243
SLE vs. Tuscany 1000G	GG + GC vs. CC	0.6736	0.3367, 1.3477	1.117	0.2641	0.4511
	G vs. C	0.6280	0.3348, 1.1779	1.450	0.1471	0.2070
RA vs. EU gnomAD	GG + GC vs. CC	1.4041	0.8753, 2.2525	1.408	0.1593	0.0820
	G vs. C	1.2048	0.7901, 1.8372	0.866	0.3867	0.3847
RA vs. EU 1000G	GG + GC vs. CC	1.1446	0.6881, 1.9038	0.520	0.6029	0.1457
	G vs. C	0.9781	0.6220, 1.5374	0.096	0.9234	0.9234
RA vs. Tuscany 1000G	GG + GC vs. CC	1.0946	0.5868, 2.0418	0.284	0.7763	0.1838
	G vs. C	0.9408	0.5437, 1.6279	0.218	0.8273	0.8273
Overall vs. EU gnomAD	GG + GC vs. CC	1.1341	0.7914, 1.6252	0.686	0.4930	0.2730
	G vs. C	1.041	0.7523, 1.4400	0.243	0.8080	0.8080
Overall vs. EU 1000G	GG + GC vs. CC	0.9245	0.6159, 1.3877	0.379	0.7047	0.1943
	G vs. C	0.8451	0.5878, 1.2150	0.909	0.3636	0.3632
Overall vs. Tuscany 1000G	GG + GC vs. CC	0.8841	0.5137, 1.5215	0.455	0.6565	0.2323
	G vs. C	0.8129	0.5034, 1.3103	0.850	0.3951	0.3946

**Table 4:** The rs1800730 mutation (*HFE*, NM_000410.4, c.193A>T, p.Ser65Cys) in the LES cohort, RA cohort and cumulative autoimmune cohort compared with the population in gnomAD and 1000G databases.

Population	Genotype	Odds Ratio				Chi-Square
		OR	95% CI	Z Score	*p* Value	*p* Value
SLE vs. EU gnomAD	TT + AT vs. AA	1.411	0.1961, 10.1504	0.342	0.7324	0.8148
	T vs. A	1.8002	0.2513, 12.8978	0.585	0.5585	0.5511
SLE vs. EU 1000G	TT + AT vs. AA	0.3783	0.0493, 2.9012	0.935	0.3497	0.7707
	T vs. A	0.4652	0.0612, 3.5367	0.739	0.4596	0.4488
SLE vs. Tuscany 1000G	TT + AT vs. AA	1.2326	0.076, 19.9955	0.147	0.8831	0.5417
	T vs. A	1.6015	0.0993, 25.8227	0.332	0.7399	0.7376
RA vs. EU gnomAD	TT + AT vs. AA	0.7821	0.0484, 12.6338	0.173	0.8626	0.7254
	T vs. A	0.7746	0.0482, 12.4571	0.180	0.8570	0.4201
RA vs. EU 1000G	TT + AT vs. AA	0.2033	0.0120, 3.4331	1.105	0.2693	0.3077
	T vs. A	0.1943	0.0116, 3.2549	1.139	0.2546	0.1150
RA vs. Tuscany 1000G	TT + AT vs. AA	0.4581	0.0184, 11.3961	0.476	0.6340	0.9721
	T vs. A	0.4606	0.0186, 11.3837	0.474	0.6357	0.3956
Overall vs. EU gnomAD	TT + AT vs. AA	0.8486	0.1185, 6.0769	0.163	0.8701	0.9784
	T vs. A	0.8348	0.1170, 5.9577	0.180	0.8570	0.8569
Overall vs. EU 1000G	TT + AT vs. AA	0.2278	0.0298, 1.7372	1.428	0.1534	0.2933
	T vs. A	0.2156	0.0285, 1.6327	1.485	0.1374	0.1023
Overall vs. Tuscany 1000G	TT + AT vs. AA	0.7413	0.0458, 11.9869	0.211	0.8330	0.9775
	T vs. A	0.7422	0.0462, 11.9333	0.210	0.8333	0.8327

## Data Availability

Data are contained within the article.
